# Genome-wide computational prediction of tandem gene arrays: application in yeasts

**DOI:** 10.1186/1471-2164-11-56

**Published:** 2010-01-21

**Authors:** Laurence Despons, Philippe V Baret, Lionel Frangeul, Véronique Leh Louis, Pascal Durrens, Jean-Luc Souciet

**Affiliations:** 1 Université de Strasbourg, CNRS UMR7156, F-67083 Strasbourg, France; 2 Université Catholique de Louvain, B-1348, Louvain-la-Neuve, Belgium; 3 Institut Pasteur, Plate-forme Intégration et analyse génomique, F-75015 Paris, France; 4 Université Bordeaux 1, CNRS UMR5800, LaBRI INRIA Bordeaux Sud-Ouest (MAGNOME), F-33405 Talence, France

## Abstract

**Background:**

This paper describes an efficient *in silico *method for detecting tandem gene arrays (TGAs) in fully sequenced and compact genomes such as those of prokaryotes or unicellular eukaryotes. The originality of this method lies in the search of protein sequence similarities in the vicinity of each coding sequence, which allows the prediction of tandem duplicated gene copies independently of their functionality.

**Results:**

Applied to nine hemiascomycete yeast genomes, this method predicts that 2% of the genes are involved in TGAs and gene relics are present in 11% of TGAs. The frequency of TGAs with degenerated gene copies means that a significant fraction of tandem duplicated genes follows the birth-and-death model of evolution. A comparison of sequence identity distributions between sets of homologous gene pairs shows that the different copies of tandem arrayed paralogs are less divergent than copies of dispersed paralogs in yeast genomes. It suggests that paralogs included in tandem structures are more recent or more subject to the gene conversion mechanism than other paralogs.

**Conclusion:**

The method reported here is a useful computational tool to provide a database of TGAs composed of functional or nonfunctional gene copies. Such a database has obvious applications in the fields of structural and comparative genomics. Notably, a detailed study of the TGA catalog will make it possible to tackle the fundamental questions of the origin and evolution of tandem gene clusters.

## Background

All genomes sequenced so far feature clusters of contiguous repeated genes (or tandem gene arrays, TGAs) that must derive from a common ancestral gene after successive duplication events. Different methods were used to achieve a systematic characterization of TGAs in eukaryotic genomes. All these methods derive from those primarily used to identify any duplicate genes in complete genomes [1-3] and take into account supplementary data concerning the chromosomal location of the detected duplicate genes. For the genome of *Arabidopsis thaliana*, the BLASTP program [4] of sequence similarity search was performed on each chromosome against itself. Then, the BLASTP results were indexed by their chromosomal physical locations to screen TGAs, allowing from 0 to 10 spacers, i.e. unrelated genes that separate two tandem duplicated copies [5]. For several genomes of vertebrates, a three-step method was applied [6,7]. An all-against-all BLASTP sequence comparison was first carried out on all protein predictions of all studied species with a view to establishing similarities. On the basis of protein similarity matrices, genes were assigned to families using Markov clustering algorithms. Genes were defined as TGAs if they belonged to the same family and were either physically adjacent or separated by a maximum number of ten intervening spacer genes. Alternative versions of this method using either BLAST and Smith-Waterman algorithms or another clustering method based on the single-linkage criterion were applied to yeast [8] and plant [9] genomes.

Here, we developed an original *in silico *method to identify in a compact genome the complete set of TGAs, including those that contain degenerated paralogous copies. These nonfunctional copies of functional genes are called pseudogenes or gene relics. The term of pseudogene designates usually a DNA segment that shows high similarity to a functional gene, but is inactive due to premature stop codons, indels or the loss of regulatory sequences. Since nonfunctionality of pseudogenes is sometimes difficult to define, Zheng and Gerstein [10] proposed a classification system of genes and pseudogenes. A "gene relic" is distinguished from a "pseudogene" by their degree of degeneration. Gene relics have gone through so many mutations of type missense, nonsense or frameshift that do not constitute an open reading frame (ORF). Pseudogenes are more similar to an ORF. They are obviously easier to detect than gene relics. As the distinction is not easy and doesn't affect the results of our method, in this paper, we will use the term "gene relic" to refer to all nonfunctional gene copies present in TGAs including pseudogenes. Though TGAs have been systematically analyzed in some genomes, the frequency of TGAs containing gene relics remains unknown. Identification of these residual duplicated copies is required in order to correctly delimit the tandem repeated unit and retrace the evolutionary history of TGAs. Whereas other existing methods used to detect TGAs take 0-10 spacers into account, our method is aimed at TGAs without spacer genes. In few cases, one spacer is authorized within a TGA. From an evolutionary point of view, the structures without intervening genes between successive paralogs should be the most recent, since they display no chromosomal rearrangement such as insertion of genes. Another distinctive feature of our method, as compared with other methods, is the incorporation of a final step of manual data curation minimizing the number of false positive TGAs.

We applied our method of analysis to the complete, assembled genome sequences of nine hemiascomycete yeasts in order to obtain a catalog of TGAs covering a large phylum of phylogenetically related species. This set of data proved useful in retracing genome evolution and notably in determining whether TGAs constitute chromosomal rearrangement hotspots for expansion and contraction of gene families. The detailed sequence analysis of the TGA flanking regions could provide better knowledge of the mechanisms at work in the apparition of these particular tandem structures. Furthermore, our TGA detection method is a helpful tool for the genome annotation. In fact, it highlights sequence similarity information between some genes and their neighbors when they are included in a TGA and helps to homogenize the annotation of these contiguous gene copies.

## Results

### Algorithm

#### 1. Score calculation

**Figure 1:**
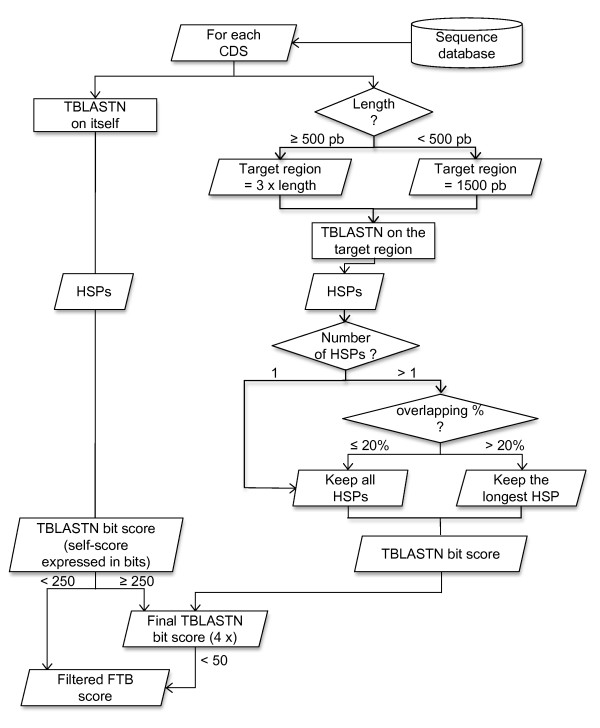
**Flowchart of the score calculation process**.

The flowchart given in figure 1 describes the score calculation process that can be divided into three principal steps.

Step1 - From each coding sequence (CDS) of a complete assembled genome, the corresponding protein sequence was compared with its upstream and downstream chromosomal regions using a TBLASTN search (expectation value threshold = 1.0). The length of the surrounding DNA sequences was three times longer than that of the CDS considered (see the Methods section). If the calculated length was below the minimal length (Lmin) of 1500 bp, the length retained was equal to Lmin. The bit score was retained for each TBLASTN sequence alignment. These initial scores, four per CDS considering its two flanking sequences and both DNA strands, were called TB (Tblastn Bit) scores. When several high-scoring segment pairs (HSPs) were obtained between a given CDS protein sequence and one of its flanking region, the total TB score was calculated as the sum of bit scores of all HSPs that correspond to the same strand (plus or minus) and do not overlap by more than 20%.

**Figure 2:**
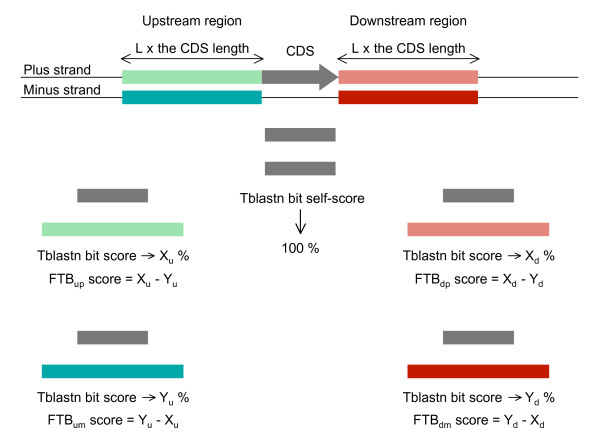
**Calculation of FTB scores**. For each CDS and its upstream and downstream surrounding regions, the X (plus strand) and Y (minus strand) values were TBLASTN bit scores expressed as a percentage of the TBLASTN bit self-score: X or Y = (bit score/bit self-score) × 100. The difference between X and Y and between Y and X were calculated to minimize the background noise that could be due to the low complexity of some DNA regions. The result is four FTB scores per CDS, two per surrounding region one of which has a value ≥ 0 and the other has the same value but ≤ 0.

Step2 - Four final scores, called FTB (Final Tblastn Bit) scores, were calculated for each CDS from the TB scores of both strands of its two surrounding sequences (Figure 2). Each TB score was first divided by a TB self-score to express it in percentage. This TB self-score was the TBLASTN bit score obtained when the protein sequence of the considered CDS was compared with its own genomic sequence. Its calculation also depended on the number and overlapping of HSPs generated by the BLAST program. The FTB scores were then obtained by subtracting two TB scores expressed as a percentage corresponding to both complementary strands of the same surrounding region.

Step3 - A filter was necessary to reduce the high background score generated by the small proteins or low sequence complexity proteins. If TB self-score < 250 and 0 < FTB score < 50, this filter was applied: filtered FTB score = FTB score - ((250 - TB self-score)/4).

#### 2. TGA extraction

**Figure 3:**
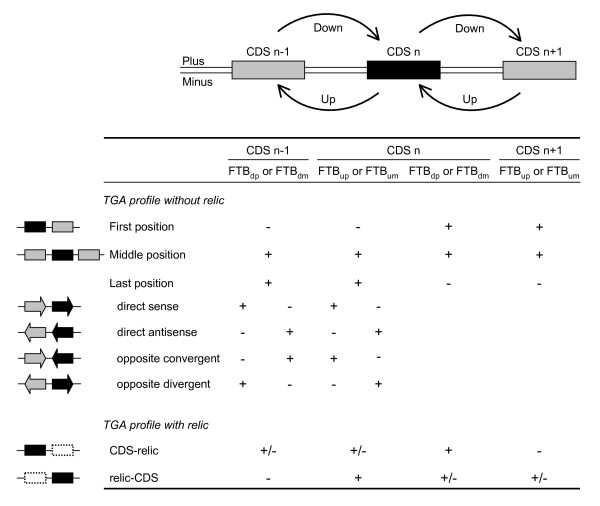
**TGA extraction**. From each CDS with at least one FTB score ≥ 10 (CDS n represented by a black box), its FTB scores and those of its two adjacent CDSs (CDSs n-1 and n+1 represented by grey boxes) are compared to decide if it belongs to a TGA and to determine its position within the TGA. CDSs constitute a TGA if their profile of FTB scores corresponds to one of those shown in the Table. The symbol + indicates an FTB score ≥ 10 and the symbol - signifies an FTB score < 10. Letters as subscript of an FTB score refer to the target sequence flanking the analyzed CDS (downstream or upstream and the plus or minus strand). Three positions within a TGA are considered for a CDS: either the CDS begins the TGA or occupies a central position or ends the TGA. The four possible orientations of two CDSs in a TGA are described when the CDS analyzed occupies the last position of the TGA. A TGA containing at least one CDS and one gene relic (represented by a grey striped box) fulfils the conditions indicated in the last two lines of the table.

All CDSs of a genome were examined, in the ascending order of their chromosomal coordinates, to select the CDSs that have at least one FTB score equal to or greater than the threshold value of 10 (for the determination of the threshold see the Methods section). To decide if a selected CDS (position n) belongs to a TGA, its own FTB scores were compared with those of its two adjacent CDSs (positions n-1 and n+1) (Figure 3). The algorithm of score comparison determines the position of each CDS within the TGA. A TGA is created when a selected CDS occupies the "first position". As long as the CDSs located downstream from this "first CDS" occupy a "middle position", the TGA is extended. But as soon as a next CDS occupies the "last position", TGA elongation comes to an end. When no directly adjacent CDS fits exactly the criteria of a tandem arrayed gene, a "relic" tag was first attributed to the CDS at position n. Then, the n-2 and n+2 CDSs were analyzed, permitting the presence of one intervening spacer gene within a TGA. The direct or opposite relative orientation of gene pairs in TGAs was determined by comparing their corresponding FTB scores (Figure 3).

#### 3. Minisatellite detection

Richard and Dujon [11] found, in the *S. cerevisiae *genome, 49 protein-coding genes containing consecutive sequence repetitions, called minisatellites, whose repeat unit size ranges from 10 bp to 192 bp. These internal tandem repeats can cause problems during TGA extraction, since some of them persist outside the coding sequence and can contribute to a FTB score ≥ 10. Moreover, in some cases, the chromosomal distance between two non-homologous genes containing minisatellites is so small that they can be related to the same TGA. Therefore, all CDSs involved in a TGA were submitted to the equicktandem (maxrepeat = 600, threshold = 20) and etandem (minrepeat = 10, maxrepeat = 300) programs of the EMBOSS package [12] to find tandem repeats in their DNA sequences. Equicktandem rapidly identifies potential repeat sizes whereas etandem searches genuine tandem repeats, calculates a consensus for the repeat block and takes gaps into account.

The research of minisatellites and the "relic" tagging in the step of TGA extraction are useful when performing the manual curation of TGAs (described in the Methods section). This enables the researcher to eliminate false positive TGAs due to short tandem repeats in gene sequences and to identify the TGAs containing gene relics, respectively.

### Implementation

Score calculation, TGA extraction and minisatellite detection are the three automated steps of our algorithm and have been implemented in Python version 2.4 http://www.python.org/. The output file of the first step is a table of the FTB scores calculated for each CDS of a given genome. From this table, another script extracts CDSs organized in tandem arrays and then identifies among these CDSs those containing minisatellites. Data of this second script can be manually analyzed to eliminate the false positive TGAs (step of manual curation). Scripts are freely available from the corresponding author who will make recommendations to users and inform them on improvements added to the computational program.

### Application: identification of TGAs in hemiascomycete yeast genomes

#### Databases

We applied our method to nine completely sequenced genomes of hemiascomycete yeasts. The *Saccharomyces cerevisiae *chromosome sequences and information on annotated genes were downloaded from Saccharomyces Genome Database (SGD) http://www.yeastgenome.org/[13]. The *Eremothecium *(*Ashbya*) *gossypii *genome sequence and its corresponding annotations were taken from Ashbya Genome Database (AGD) http://agd.vital-it.ch/Ashbya_gossypii/index.html/[14]. Génolevures website http://cbi.labri.fr/Genolevures/[15] provided all the data relative to complete genome sequences of the species *Candida glabrata*, *Debaryomyces hansenii*, *Kluyveromyces lactis*, *Kluyveromyces thermotolerans*, *Saccharomyces kluyveri *(genome sequenced in collaboration with Mark Johnston, Washington University Department of Genetics), *Yarrowia lipolytica *and *Zygosaccharomyces rouxii*.

#### Computation of all TGAs in the nine yeast genomes

**Figure 4:**
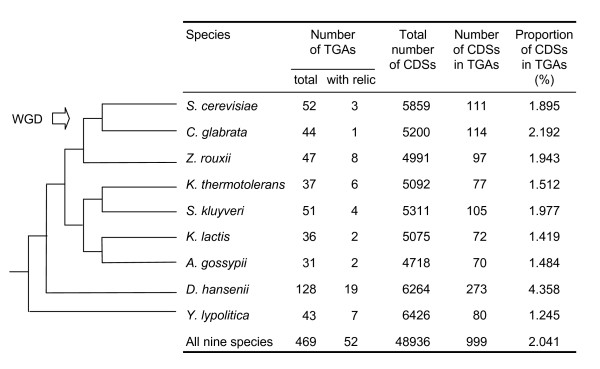
**Identification of TGAs in hemiascomycete yeast genomes**. The phylogram of the nine species considered is adapted from reference [41]. The whole genome duplication (WGD) event occurred before the divergence of *S. cerevisiae *and *C. glabrata*. The number of CDSs refers to the total number of annotated coding sequences taken into account when searching TGAs in each genome. All tandem arrays consisting of at least one CDS and one gene relic are counted as "TGAs with relic".

We define a TGA as a structure of contiguous paralogous gene copies, which are either functional or degenerated copies (gene relics), allowing for the presence of one heterologous gene inserted between the two homologous copies. In accordance with this definition, we developed an *in silico *method to detect such TGAs in complete eukaryotic genomes. This method was applied to nine hemiascomycete yeast genomes. 469 TGAs comprising 999 CDSs were identified in this set of genomic data, which represents 2% of CDSs involved in TGAs in relation to the total number of CDSs present in these nine genomes (Figure 4 and Additional file 1). This proportion is independent of the phylogenetic position of species and is not higher in the post-WGD (whole genome duplication) species *S. cerevisiae *and *C. glabrata*. Interestingly, the *D. hansenii *genome shows a percentage of CDSs in TGAs twice higher than the other species. Another remarkable result is the percentage of TGAs that include a duplicated gene relic (11%).

On an average 6% of yeast genes contain intron sequences, generally only one intron per gene. Among all CDSs that belong to TGAs, 2.5% (25 CDSs in 16 TGAs) have an intron. In half of the 16 TGAs concerned, an intron was not present in all the duplicated copies. The physical distribution of TGAs is relatively homogeneous along the different chromosomes of each species (data not shown).

A first assessment of functional bias for genes in tandem arrays was based on Gene Ontology (GO) term annotation information [16]. Functions such as cellular homeostasis, cell wall organization and biogenesis, conjugation and response to stress, are overrepresented in TGAs. The proportion of genes involved in TGAs is very low in the following functional classes: transcription, translation, RNA metabolic process and ribosome biogenesis and assembly (data not shown).

#### Distributions of TGA sizes and CDS orientations in TGAs

**Figure 5:**
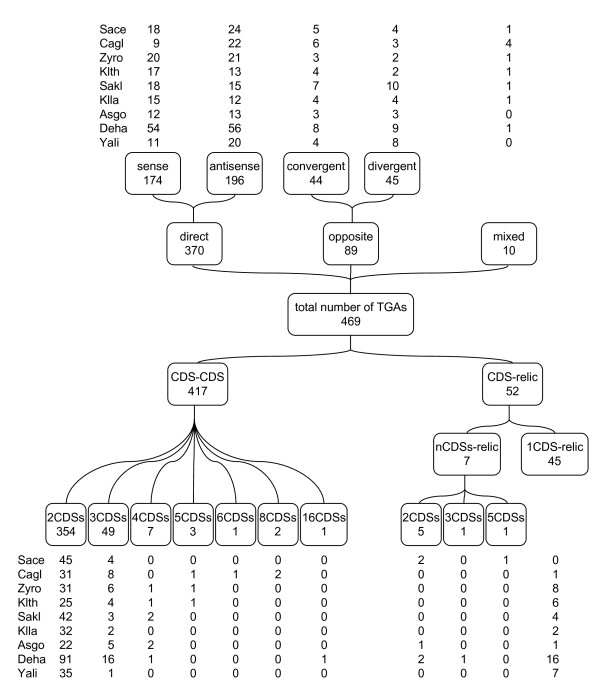
**Distribution of TGAs according to the number and orientation of their constituent CDSs**. The 469 TGAs are distributed among the nine yeast species studied. The direct orientation refers to TGAs in which all CDSs share the same orientation (sense => => or antisense <= <=). When TGAs are composed of two CDSs located on different DNA strands (convergent => <= or divergent <= =>), their orientation is called "opposite". Mixed TGAs contain at least one CDS pair in direct orientation and one CDS pair in opposite orientation. Sace: *S. cerevisiae*, Cagl: *C. glabrata*, Zyro: *Z. rouxii*, Klth: *K. thermotolerans*, Sakl: *S. kluyveri*, Klla: *K. lactis*, Asgo: *A. gossypii*, Deha: *D. hansenii *and Yali: *Y. lipolytica*.

TGAs were classified according to the number of CDSs they contain. Among the nine yeast genomes, the size of TGAs ranged from one CDS to 16 CDSs (Figure 5). TGAs belonging to the 1CDS-relic class consist of one CDS and one gene relic and represents ~10% of the total number of TGAs, a proportion equivalent to that of the 3CDSs-TGA class. The majority of TGAs (76.5%) comprise two CDS copies and the percentage in four copies falls to 1.5. Large TGAs of six and eight CDSs (one and two arrays, respectively) are concentrated in *C. glabrata*, but the largest one containing 16 CDSs is in *D. hansenii*. The latter (TGA n°234), located on the chromosome E of *D. hansenii*, has been manually reconstructed since it was disrupted 3 times by heterologous genes. In the sequenced strain of *S. cerevisiae*, S288C, the TGA size does not exceed five CDSs and the 1CDS-relic class of TGAs is absent. Nevertheless, three TGAs made up of more than one CDS plus one relic (nCDSs-relic class) are present in this reference strain.

CDSs in TGAs are principally in a direct orientation (78.9%) with approximately the same proportion of sense and antisense orientations (Figure 5). In *C. glabrata *TGAs, the antisense orientation is represented 2.4 times more than the sense orientation, although the number of CDSs on the minus strand (2649 CDSs) and on the plus strand (2551 CDSs) is identical. TGAs consisting of two CDSs oppositely oriented represent 19.0% of total TGAs. The low frequency of mixed TGAs (2.1%) is in correlation with the low percentage of TGAs containing at least 3 CDSs (13.9%) and of which at least two copies have a convergent or divergent orientation, this being the less frequent orientation of a CDS pair (expected frequency = 0.190 × 0.139 = 0.026).

#### Data curation

The originality of our method of TGAs detection lies in the possibility to identify TGAs containing degenerated gene copies. From the nine yeast genomes analyzed, two different sets of CDSs, possibly close to a gene relic, appeared during the TGA extraction step and received a relic tag: (i) 360 isolated CDSs with at least one FTB score ≥ 10 and (ii) 23 CDSs, located on one extremity of a TGA comprising at least two CDS, with two FTB scores ≥ 10 (see Additional file 2). 15% (53 CDSs) and 22% (5 CDSs) of these two respective sets of tagged CDSs were first retrieved by an automated curation step to create new TGAs. After this procedure, a manual data curation produced supplementary TGAs or some TGAs recomposed from 24% (85 CDSs) and 48% (11 CDSs) of the two respective sets of tagged CDSs. No false positive CDS was found among the 23 tagged CDSs included in a TGA, whereas approximately half the isolated CDSs (177 false positives) were eliminated. Finally, 52 CDSs were located near a homologous gene relic: 45 constitute the class of TGAs that contain just one CDS (1CDS-relic) and 7 belong to larger TGAs (nCDSs-relic).

A possible source of false positive TGAs is the presence of minisatellites in the coding sequences. Among the 999 CDSs implicated in a TGA, 199 (20%) have internal tandem repeats (see Additional file 2). The manual examination of these suspicious CDSs revealed that 6.6% of the total number of TGAs before curation (33/502) were false positives. Nevertheless, it was difficult in some cases to decide whether the similarity between contiguous CDSs is partially or totally due to minisatellites. This problem occurred when long stretches of tandem repeats covered a large part of the CDSs. Therefore, the percentage of false positives is probably slightly underestimated.

#### Distributions of FTB scores

**Figure 6:**
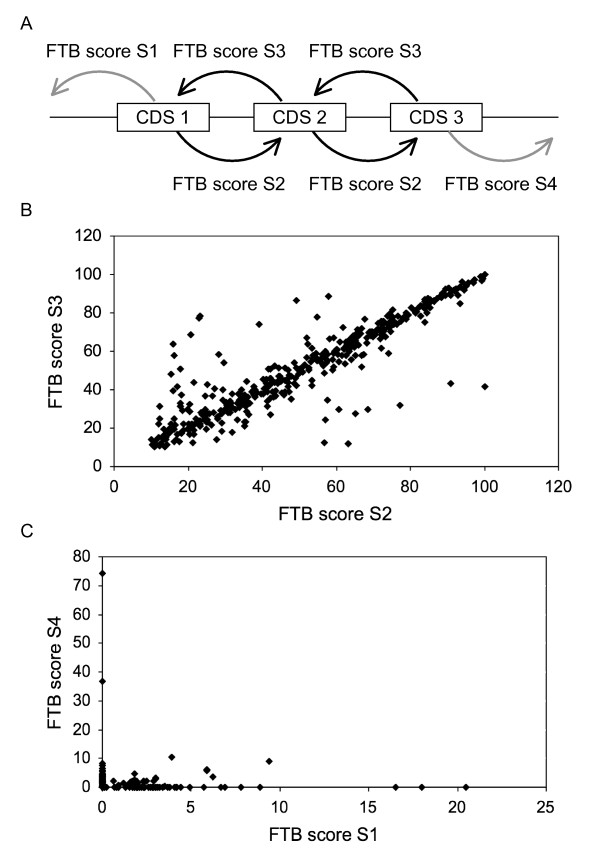
**Comparisons of FTB score pairs**. (A) The pairs of FTB scores considered per TGA. Only TGAs not manually corrected with more than one CDS (349 TGAs in total for the 9 genomes) were retained for the analysis. For each of these TGAs, S2 and S3 correspond to FTB scores ≥ 10 associated with each pair of duplicated CDSs. Whereas S1 and S4 are FTB scores ≥ 0 of CDSs located at the extremities of the TGA. S1 concerns the region upstream from the TGA and S4 the region downstream. (B) Comparison of S2/S3 score pairs. (C) Comparison of S1/S4 score pairs.

The degree of gene conservation in a TGA can be estimated by comparing BLAST bit scores between two genes. FTB scores were calculated from the result of TBLASTN sequence alignments. Two FTB scores ≥ 10 (S2 and S3) were obtained per gene pairs in a TGA. Two additional FTB scores (S1 and S4) characterize both chromosomal regions surrounding each TGA. The TGA flanking regions can contain CDSs, but these coding sequences have no homology with members of the TGA (Figure 6A). For each pair of tandem repeated CDSs (TGP for tandem gene pair), except those manually recomposed, the FTB score S2 was compared with the FTB score S3 (Figure 6B). These two scores are mostly equal or equivalent. In 6.2% of cases, the difference between both scores is > 20 and is due principally (60% of cases) to the size difference between both genes of a TGP. The comparison between the FTB scores S1 and S4 shows that in 93% of cases (323 TGAs) at least one of these two scores is equal to 0 (Figure 6C). Few TGAs (6 in total) have one score, S1 or S4, greater than 10. They correspond to TGAs that start or end with a homologous gene relic. Therefore, they belong to the nCDSs-relic class of TGAs. One of the seven TGAs in this class is not represented here because it was corrected manually.

#### Evolution of TGAs in comparison with the one of the other duplicated genes

**Figure 7:**
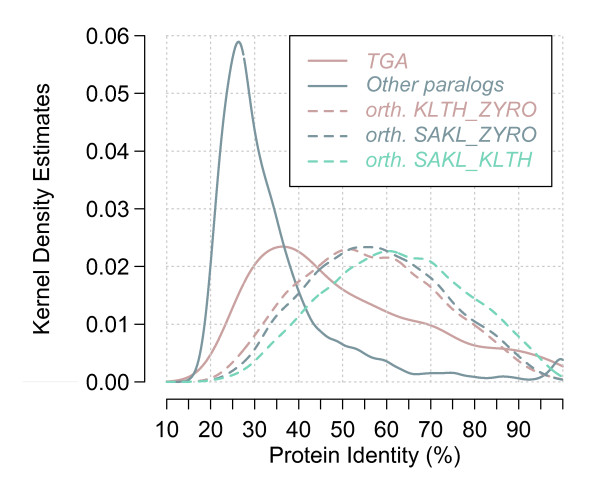
**Distribution of protein identities between pairs of homologous genes**. Protein identity distribution was computed for pairs of homologs from BLASTP comparisons (see Methods) and plotted as a kernel density estimation plot. Average distributions for the nine yeast species analyzed were calculated in both cases of TGA members and other paralogous genes. All pairwise alignments between two species were performed for orthologs, but only the distributions calculated from three species were plotted as examples (the other ones showing a similar profile). KLTH: *K. thermotolerans*, ZYRO: *Z. *rouxii and SAKL: *S. kluyveri*.

Distribution of amino acid sequence identities between pairs of homologous proteins was computed to evaluate the divergence between paralogous (tandem arrayed and dispersed) and orthologous gene copies (Figure 7). Orthologous proteins show monomodal distributions and average identities comprised between 48-58%. They represent the most sequence-conserved homologous proteins. The distribution of tandem paralogs is intermediate between the distribution of other paralogs and the distribution of orthologs. Thus, paralogs in TGAs are less divergent than dispersed paralogs. Although the global distributions of these two sets of paralogs are not bimodal, the protein identities do not follow a normal distribution since the paralogs exhibiting high sequence conservation are found more frequently than their poorly conserved counterparts. This feature is amplified in the case of tandem duplicated genes.

## Discussion

The sequence homology between tandem duplicated genes favors recombination events, followed by the deletion or deletion-fusion of some copies. Mutational events can also cause the disappearance of genes in TGAs but more progressively. This death of genes is compensated by the maintenance of some other copies and/or the creation of new gene copies to ensure the original gene function [1] (for review, see [17]). Therefore, a certain number of TGAs must conserve the trace of these phantom gene copies called pseudogenes or gene relics depending on their degree of degeneration [18-20]. All the methods used so far to detect TGAs in complete genomes were based on the search of intact gene copies that are homologous by sequence similarity and are organized in tandem repeats [5-9]. Therefore, the proportion of TGAs containing degenerated gene copies was still unknown. We developed a novel computational method to predict all TGAs, including the ones with gene relics, in eukaryotic genomes. This methodological approach is relevant when we know that human and other eukaryotic genomes contain thousands of pseudogenes [21]. In the *Saccharomyces cerevisiae *genome, the pseudogene number was reported by different sources and a compilation of these data, giving 278 pseudogenes, was presented by Lafontaine *et al. *[22]. Then, the pseudogene frequency in the yeast genome is approximately of one pseudogene/21 CDSs (in reference to 5823 annotated CDSs in total). Our method does not perform pairwise BLASTP comparisons of gene sequences to detect similarities, but compares each coding sequence with the DNA sequences of its two surrounding chromosomal regions using the TBLASTN program. The detection of sequence similarity between a CDS and an adjacent non-coding region reveals the putative presence of a CDS-relic tandem repeat. In order to verify the existence of a relic in such repeats, a manual curation was performed after TGA extraction.

Our method is efficient for species that have a compact genome such as prokaryotes and unicellular eukaryotes. Its adaptation to multicellular eukaryotic species should take into account the physical characteristics of their genomes. In fact, the identification of tandem repeated gene copies using TBLASTN could be difficult with large genomes that feature much longer intergenic regions and coding sequences with frequent and long introns especially if they are subjected to alternative splicing. Moreover, short DNA repeats and notably minisatellites are more frequent in complex genomes especially in intergenic sequences (for review, see [23]). But, these repeated sequences are mainly located in intergenic regions and will not represent an issue as our method detects minisatellites only within the DNA sequence of tandem repeated genes. Despite these limitations, we proceeded with a first evaluation of the proposed method on the chromosome 4 of the plant *Arabidopsis thaliana*. 223 TGAs and 145 isolated CDSs potentially in tandem with a gene relic were detected by using the same parameters than those used for yeast genomes. Although no manual curation was performed on these data, they agree with the results previously obtained predicting 196 TGAs (no spacer) or 250 TGAs (one spacer authorized) on the *A. thaliana *chromosome 4 [5]. If confirmed after manual curation (please note that for yeast application, the manual curation reduced by half the number of "one CDS - one gene relic" TGAs), these first results illustrate the interest of the method for the identification of TGAs including the "one gene and one gene relic" arrays.

We applied our method to nine annotated complete genomes of hemiascomycete yeasts. Results show that, on an average, 2% of CDSs are included in TGAs and 11% of these TGAs contain a gene relic. The percentage of tandem arrayed genes in the yeast genomes is lower than the one calculated for other eukaryotic species. For two plant genomes, 9.3% and 13.4% of genes belong to TGAs having 0 or 1 spacer gene and this value rises to 15.5% if 10 spacers are allowed [9]. Variation from 9.0% to 21.4% (0 or 1 spacer per TGA) was reported in the case of 11 vertebrate species [7]. The total number of TGAs had already been estimated for five yeast genomes by another method based on protein families and authorizing 0-10 spacers [8]. Among these five genomes, only those of *S. cerevisiae*, *C. glabrata*, *K. lactis *and *Y. lipolytica *are exploitable for a comparative analysis of TGA data since Dujon et *al. *[8] used another version of the *D. hansenii *genome. Since 2004, this genome was reassembled and its re-annotation showed that the new sequence is quite different compared to the preceding version, notably with regard to the repeated regions (B. Dujon, unpublished data). On the 501 genes constituting tandem arrays (229 TGAs) in the four yeast species, 47% (109 TGAs) were detected by both methods, 28% (66 TGAs) were specifically identified by our method and 25% (54 TGAs) were found only by the Dujon et *al. *[8] method. The detailed analysis of the non-common genes highlights the specificities of the two methodological approaches. Some TGAs are not found by our method for two main raisons: (i) more than one spacer gene is present between two tandem duplicated genes (80% of cases), and (ii) the distance between two tandem genes is longer than the average (20%). Concerning the method used by Dujon *et al. *[8], three points explain the absence of a certain number of TGAs: (i) the presence of gene relics in tandem repeats (5% of cases), (ii) the presence of minisatellites in protein sequences (43%), and (iii) genes of the same TGA belonging to different protein families (52% of cases, points (ii) and (iii) are sometimes associated). In conclusion, each method has its weaknesses and strengths. The main advantages of the method we propose are that we have no requirement in terms of protein assignment to families and our ability to detect dead gene copies and minisatellites in TGAs.

*D. hansenii *features the highest proportion (4.4%) of tandem repeated genes among the nine yeasts analyzed. The mechanism of tandem gene duplication must be particularly efficient in this cryo- and osmotolerant marine species, probably in response to environmental constraints. The majority of TGAs in yeast genomes (76.5%) are the size of two CDSs and CDS orientation is mainly direct (~80% of TGAs have CDSs on the same strand). These characteristics are also observed in other species. The largest TGAs are found in *C. glabrata *(two arrays of 8 CDSs encoding proteins similar to aspartic proteases and mannosyltransferases, respectively) and in *D. hansenii *(one array of 16 CDSs without similarity). Strong selective pressure may have intervened to maintain the size of these large TGAs, despite the fact that they represent selected targets for chromosomal rearrangements via recombination events.

TGAs comprising gene relics were identified in the nine yeast genomes, either as 1CDS-relic (most cases) or nCDSs-relic TGAs. Their frequency, equal to 11.1%, is approximately identical to that of 3CDSs-TGAs (10.4%). This significant figure confirms that an important fraction of TGAs have genes that have not evolved in a concerted fashion but comply with the birth-and-death mechanism of evolution. In fact, TGAs are thought to evolve according to two major models [24]. Concerted evolution homogenizes the duplicated gene sequences to preserve the gene function and serves to increase gene dosage. In contrast, in the birth-and-death evolutionary model, sequence divergence within a TGA favors the emergence of new functions but also gene inactivation or disappearance. This second model is consistent with the role of TGA formation in rapid adaptation to environmental necessities. Interestingly and similarly, the results of our functional analysis (based on comparison of GO term annotations) show that biological processes related to cell-environment relationships (such as cell wall biogenesis and response to stress) are overrepresented for genes in TGAs as compared with other genes.

The step of manual curation was pertinent since it enabled the authors to: (i) eliminate 49% of false positive CDSs displaying a "relic" tag (tagged CDSs), (ii) retrieve 25% of tagged CDSs to create new TGAs or complete pre-existent TGAs and (iii) eliminate 6.6% of false positive TGAs containing CDSs with internal minisatellites. Applied to genomes having a total number of genes higher than the yeast genomes, our method would probably produce a number of tagged CDSs so great that their manual verification would become unachievable. In this case, a modification of the threshold value of FTB score used to extract TGAs could be envisaged to reduce the number of false positive CDSs. For the global analysis of all nine yeast genomes, an increase of this parameter from 10 to 15 drastically decreased the background noise (61% diminution in the number of tagged CDSs, from 307 to 120), without greatly modifying the number of detected TGAs (8.1% loss, from 356 TGAs to 327). The length of the sequences flanking a CDS, determined as a number of times the length of the CDS (n × L_CDS_) and used as a target for the similarity search, is another variable parameter. But its modification must be limited in a given species since it depends on the size of the coding genes and on overall gene density.

The analysis of the FTB score distribution in TGPs was performed to evaluate more precisely the general evolutionary process of TGAs. A bimodal repartition is expected as one part of TGAs is supposed to have undergone neofunctionalization or pseudogenization, the other part representing sequence-conserved TGAs. The scatter plot given in the results (Figure 6B) does not show a profile characteristic of a bimodal distribution. This observation could mean that TGAs follow a continuous process of degeneration.

On the other hand, we compared protein identity distributions between different sets of homologous gene pairs. Results show that paralogs in TGAs have, on an average, amino acid sequences better conserved than dispersed paralogs, but are more divergent than orthologs. Gene conversion is a plausible mechanism for sequence homogenization among many tandem gene repeats and it is reported that its frequency is lower between separated gene copies [25-27]. Another alternative is that tandem duplicate genes supposedly arise from more recent events of gene duplication than dispersed duplicate genes. The mechanism of tandem gene duplication usually tends to respond more rapidly to environmental stimuli. It has recently been reported that this mechanism has generated ~80% of the new genes specific to *Drosophila *species [28]. For nascent gene copies shared by multiple *Drosophila *species, 44% and 34% are dispersed and tandem repeated duplicates, respectively. Moreover, Zhou *et al. *[28] have found that tandem duplicate genes have on an average younger ages but lower survivorships as against dispersed genes. Along the same lines, Leh-Louis *et al. *[29] observed that the largest TGA (five CDSs) in *S. cerevisiae *ends with a relic and its size varies extensively among different strains of the same species.

## Conclusions

In many comparative genomics studies, large-scale detection of tandem duplicated genes is a necessary step to tackle basic questions about gene duplication, notably the mechanisms of TGA formation and evolution. First, the extension of TGAs, such as ribosomal DNA gene repeats, is mainly explained by mitotic or meiotic events of unequal sister-chromatid crossover [30,31]. But there is no convincing mechanism to explain the transition from one original copy to two tandem repeated copies. Second, chimeric or truncated genes are often observed at the edges of segmental duplications [32-34] and retroposition-mediated single gene duplications [35-37], although their presence at the borders of TGAs is not reported. Gene limits could be recognized by the mechanism of tandem gene duplication. Third, sequence divergence frequently observed between genes sharing the same tandem array suggests that selective pressures are applied to such areas, favoring the emergence of new functions. TGAs would be breeding grounds for new genes. We will address these interesting questions with a detailed analysis of the TGA catalog produced by our computational prediction method from hemiascomycete yeast genomes. Different methods were used to predict in a genome the complete set of TGAs. Nevertheless, the novelty and advantage of our method compared with existing methods lies in its detection of additional TGAs containing degenerated paralogous gene copies. Identification of these nonfunctional copies of functional genes is a crucial stage to determine the borders of the repeated unit and retrace the evolutionary history of tandem arrayed paralogs. Therefore, the size of tandem repeated units in all yeast genomes analyzed will be correctly determined and chimeric or truncated genes will be searched at the extremities of these units. We will also identify the possible presence of pertinent sequence elements flanking TGAs (such as transposon sequences, replication origins and small conserved DNA motifs) in order to define mechanism(s) of TGA formation. Finally, multiple sequence alignments will be used to establish phylogenies and calculate nucleotide substitution rates in both silent and non-silent positions in order to date the successive events that have occurred at tandem repeated loci and identify the evolutionary forces (mutation/selection).

## Methods

### Manual curation

To obtain high quality data, we performed a manual curation of the results produced by the TGA extraction method. All CDSs showing a "relic" tag, i.e. with FTB score(s) ≥ 10 but no neighboring CDS (at positions n +/- 1 and n +/- 2) with a correct profile of FTB scores (Figure 3), were submitted to a dotplot analysis. Their genomic sequences were compared pairwise with their surrounding region where a significant FTB score was found, using the DNA Strider™ 1.4 dot matrix (stringency = 15, window = 23) [38]. This approach allowed us to detect sequence similarity in intergenic regions through the visual perception of diagonal lines. When a significant diagonal stood out against the background noise, we considered that the intergenic region analyzed contained a gene relic tandem arrayed with the tagged CDS. The same dotplot approach was performed with all CDSs that gave a score > 0 with the equicktandem or etandem program. The presence of minisatellites in these coding sequences was confirmed by protein self-matrices (stringency = 5, window = 23). CDSs with confirmed internal repeats were aligned with the other CDSs that belong to the same TGA using protein-against-protein matrices, in order to verify if the high FTB scores related to these TGAs were not only due to minisatellites.

### Impact of two parameters on the amounts of TGAs and tagged CDSs detected

Two parameters mainly influence the results of our TGA detection method: (i) the length of the DNA sequences surrounding each CDS of a given genome and in which BLAST will look for an homology and (ii) the threshold of FTB score used to extract TGAs. The first parameter is a multiple of the length of the CDS considered (n × L_CDS_; most cases) or is equal to the minimal length of 1500 bp. In view of the size of protein coding genes (1.38 kb) and the overall gene density (one gene per 2 kb) in the yeast genomes [8], a length of genomic sequences of 2-3 times the CDS size seems to be an interval of correct values to find the tandem duplicated copies. Preliminary tests performed on the yeast genome of *D. hansenii *allowed us to define the limiting values of the FTB score threshold (10 ≤ threshold ≤ 15) and thus obtain a good compromise between a high number of detected TGAs and a low background noise which basically is assessed as the number of CDSs with a relic tag. We measured the real impact of the modification of these two parameters on the counts of TGAs extracted and tagged CDSs (see Additional file 3). The number of tagged CDSs, obtained before manual curation, is mostly influenced by the threshold parameter. Modification of only this parameter, from 10 to 15 or inversely, induces a variation of 59% or 61% in the number of the tagged CDSs, whereas a constant threshold and a variable length result in a smaller variation of 13% or 18%. The number of TGAs is less influenced (7.9-10.7% variation) than the number of tagged CDSs (13-61% variation). Modification of only one parameter (n × L_CDS _or threshold) leads to a variation percentage of the TGA number that does not exceed 10.7%. Therefore, both parameters must be modified simultaneously to obtain the highest variation of the TGA number (18%). We chose to use the combination threshold = 10 and length = 3 × L_CDS _in order to identify the maximum number of TGAs, although the background noise was also maximum and then required a more important manual verification of tagged CDSs. Under these parameter conditions, the false negative TGA rate was estimated to be less than 8%.

### Evolutionary analysis of TGAs, other paralogs and orthologs

The paralogous gene copies of the nine yeast genomes analyzed were defined from the protein families clustered by the Nikolski and Sherman [39] method. In a given species, all paralogous genes were identified. The members of different TGAs are subsets of these paralogous genes. The orthologous gene pairs between these same species were determined from SONS (Subset of Orthologs defined by Neighborhood and Similarity). Two genes of different species belong to the same SONS if their products are members of the same protein family and if they share at least one pair of homologous neighboring genes, i.e. if synteny is conserved [40].

Amino acid identities between the products of two homologous genes (paralogs or orthologs) were calculated from BLASTP alignments without low complexity filter. For paralogs of a given family in a given species, the identity score was calculated on a single pairwise comparison randomly sampled among all possible pairwise comparisons between paralogs. Indeed, if all pairwise comparisons had been achieved, it would have biased the distribution in favor of the largest family of paralogs. In the case of tandem arrayed paralogs, each TGA member (except gene relics without corresponding protein) was aligned against all other members. For orthologs, protein identity distribution was computed from all pairwise alignments of SONS orthologs determined between two species.

## Abbreviations

CDSs: coding sequences; TGAs: tandem gene arrays; TGPs: tandem gene pairs.

## Authors' contributions

JLS, LF and LD conceived of the project. VLL and LF performed preliminary tests on yeast genomes to determine parameters of the TGA detection method. LD developed the Python computer program that predicts TGAs, performed the genome-wide analysis and wrote the manuscript. PVB assisted in analyzing the data and preparing the paper. PD contributed comments and suggestions. All authors read and approved the final manuscript.

## Supplementary Material

Additional file 1 **Tabular data S1 - List of CDSs that constitute TGAs in the nine hemiascomycete yeast genomes**. Additional data file 1 is a Microsoft Excel spreadsheet (.xls) containing all the identified tandemly arrayed genes in the nine yeast genomes analyzed. All genetic elements were designated using a systematic nomenclature system adopted in the "Génolevures" projects [42], except for YAR062W which is a pseudogene (TGA n°49). Chromosomal coordinates are indicated for each gene locus. Click here for file

Additional file 2 **Table S1 - Impact of the data curation steps on the count of TGAs**. Three steps of data curation were performed to distinguish between CDSs belonging to a TGA and false positive CDSs: first, an automated curation of tagged CDSs (with a "relic" tag) analyzing the FTB score value of CDSs at positions n+2 and n-2, second, a manual curation of the remaining tagged CDSs and finally, a manual curation of CDSs in which minisatellites were detected by the equicktandem or etandem EMBOSS program. Click here for file

Additional file 3 **Table S2 - Parameters influencing the results of the in silico TGA detection method**. We measured the influence of two parameters on the number of TGAs and tagged CDSs identified before the step of manual data curation. The first parameter intervening in the score calculation step is the length of the chromosomal regions located upstream and downstream from each CDS (n times as long as the CDS length). The threshold value of FTB score is the second parameter used to select CDSs belonging to a TGA during the TGA extraction step. Click here for file
