# Supplementary material for: Genome-wide computational prediction of tandem gene arrays: application in yeasts
Source: BMC Genomics. 2010 Jan 21;11:56. doi: 10.1186/1471-2164-11-56 (PMC2822764; doi:10.1186/1471-2164-11-56)
Supplement: Additional file 2 — Table S1 - Impact of the data curation steps on the count of TGAs. Three steps of data curation were performed to distinguish between CDSs belonging to a TGA and false positive CDSs: first, an automated curation of tagged CDSs (with a "relic" tag) analyzing the FTB score value of CDSs at positions n+2 and n-2, second, a manual curation of the remaining tagged CDSs and finally, a manual curation of CDSs in which minisatellites were detected by the equicktandem or etandem EMBOSS program. [file 1471-2164-11-56-S2.PDF]

Table S1: Impact of the data curation steps on the count of TGAs.

Species	CDS with a relic tag									CDS with minisatellites	
	not included in a TGA[a]					included in a TGA[b]					
	Number of CDSs before curation	Automated curation[c]	Manual curation[d]	Number of false positive CDSs	Number of CDSs after curation[e]	Number of CDSs before curation	Automated curation[c]	Manual curation[d]	Number of CDSs after curation[f]	Number of CDSs before manual curation	Number of TGAs eliminated[g]
S. cerevisiae	45	10	18	17	0	4	0	1	3	21	0
C. glabrata	22	8	1	12	1	3	0	3	0	39	3
Z. rouxii	32	2	5	17	8	2	0	2	0	15	3
K. thermotolerans	29	2	7	14	6	2	0	2	0	14	3
S. kluyveri	47	5	8	30	4	1	1	0	0	23	8
K. lactis	26	4	7	13	2	0	0	0	0	11	1
A. gossypii	29	1	3	24	1	2	1	0	1	14	2
D. hansenii	74	17	25	16	16	9	3	3	3	37	1
Y. lypolitica	56	4	11	34	7	0	0	0	0	25	12
All nine species	360	53	85	177	45	23	5	11	7	199	33

[a]CDS at position n with at least one FTB score ≥ 10 but both CDSs at positions n+1 and n–1 have not the score profile defined for a TGA.
[b]CDS at position n with two FTB scores ≥ 10 but just one CDS at position n+1 or n–1 has the "TGA profile".
[c]Number of tagged CDSs automatically retrieved and included in a new or pre-existent TGA since at least one CDS at position n+2 or n–2 has the "TGA profile".
[d]Number of tagged CDSs manually retrieved and included in a new or pre-existent TGA.
[e]This count finally gives the number of CDSs that belong to the 1CDS-relic class of TGAs.
[f]Number of CDSs that belong to a TGA containing at least two CDSs and are adjacent to a degenerated duplicated copy (nCDSs-relic class).
[g]TGAs manually removed because the high value of their corresponding FTB scores was only due to the presence of minisatellites in CDS sequences.
