# Supplementary material for: Genome-wide computational prediction of tandem gene arrays: application in yeasts
Source: BMC Genomics. 2010 Jan 21;11:56. doi: 10.1186/1471-2164-11-56 (PMC2822764; doi:10.1186/1471-2164-11-56)
Supplement: Additional file 3 — Table S2 - Parameters influencing the results of the in silico TGA detection method. We measured the influence of two parameters on the number of TGAs and tagged CDSs identified before the step of manual data curation. The first parameter intervening in the score calculation step is the length of the chromosomal regions located upstream and downstream from each CDS (n times as long as the CDS length). The threshold value of FTB score is the second parameter used to select CDSs belonging to a TGA during the TGA extraction step. [file 1471-2164-11-56-S3.PDF]

Table S2: Parameters influencing the results of the *in silico* TGA detection method.

Species	L_{CDS} X 3 threshold = 10		L_{CDS} X 3 threshold = 15		L_{CDS} X 2 threshold = 10		L_{CDS} X 2 threshold = 15	
	Number of TGAs	Number of tagged CDSs[a]	Number of TGAs	Number of tagged CDSs	Number of TGAs	Number of tagged CDSs	Number of TGAs	Number of tagged CDSs
S. cerevisiae	35	35	35	16	34	21	33	6
C. glabrata	43	14	41	4	36	16	32	10
Z. rouxii	35	30	31	13	32	21	28	9
K. thermotolerans	27	27	25	12	28	18	24	8
S. kluyveri	40	42	36	13	36	38	32	13
K. lactis	28	22	24	7	25	20	21	7
A. gossypii	29	28	26	7	28	26	24	6
D. hansenii	95	57	88	32	89	49	83	28
Y. lypolitica	24	52	21	16	20	42	16	17
All nine species[b]	356	307	327 (-8.1 %)	120 (-61 %)	328 (-7.9 %)	251 (-18 %)	293 (-18 %)	104 (-66 %)

[a]Number of CDSs with a relic tag.
[b]The percentage in brackets indicates the proportion of lost TGAs or tagged CDSs according to the sum obtained with the reference parameters L_{CDS} X 3 and threshold = 10.
